# New Stability Indicating RP-HPLC Method for the Estimation of Cefpirome Sulphate in Bulk and Pharmaceutical Dosage Forms

**DOI:** 10.3797/scipharm.1104-25

**Published:** 2011-08-07

**Authors:** Kareti Srinivasa Rao, Keshar Nargesh Kumar, Datta Joydeep

**Affiliations:** Roland Institute of Pharmaceutical Sciences, Berhampur, Orissa-760 010, India

**Keywords:** Cefpirome sulfate, Stability studies, Assay, Validation

## Abstract

A simple stability indicating reversed-phase HPLC method was developed and subsequently validated for estimation of Cefpirome sulphate (CPS) present in pharmaceutical dosage forms. The proposed RP-HPLC method utilizes a LiChroCART-Lichrosphere100, C18 RP column (250 mm × 4mm × 5 μm) in an isocratic separation mode with mobile phase consisting of methanol and water in the proportion of 50:50 % (v/v), at a flow rate 1ml/min, and the effluent was monitored at 270 nm. The retention time of CPS was 2.733 min and its formulation was exposed to acidic, alkaline, photolytic, thermal and oxidative stress conditions, and the stressed samples were analyzed by the proposed method. The described method was linear over a range of 0.5–200μg/ml. The percentage recovery was 99.46. F-test and t-test at 95% confidence level were used to check the intermediate precision data obtained under different experimental setups; the calculated value was found to be less than the critical value.

## Introduction

**Fig. 1:**
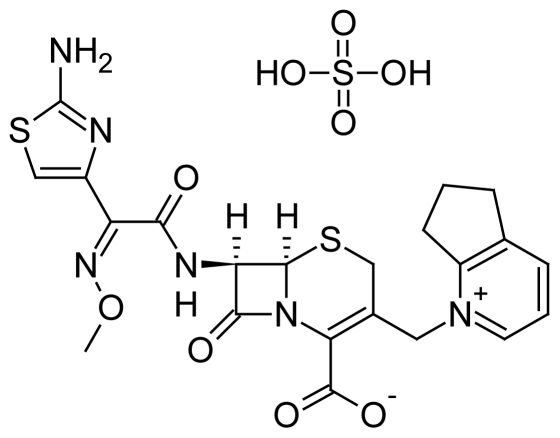
Cefpirome Sulphate

Cefpirome is chemically (6*R*,7*R*)-7-{[(2*Z*)-2-(2-amino-1,3-thiazol-4-yl)-2-(methoxyimino)-acetyl]amino}-3-(6,7-dihydro-5*H*-cyclopenta[*b*]pyridinium-1-ylmethyl)-8-oxo-5-thia-1-aza-bicyclo[4.2.0]oct-2-ene-2-carboxylate (Figure 1). It is a broad-spectrum semisynthetic β-lactamase resistant fourth generation cephalosporin bearing a quaternary ammonium group at the 3 position of the cephem nucleus. It is used for the treatment of upper and lower urinary tract as well aslower respiratory tract, skin and soft tissue infections. Cefpirome is excreted largely unchanged in the urine with a half-life of 2 hours [1]. It has an expanded spectrum of activity against Pseudomonas sp., enterococci, and staphylococci, as well as other gram-positive and gram-negative bacteria [2].

Forced degradation or stress testing is undertaken to demonstrate specificity when developing stability-indicating methods, particularly when little information is available about potential degradation products. The ICH guideline entitled “Stability Testing of New Drug Substances and Products” requires the stress testing to be carried out to elucidate the inherent stability characteristics of the active substances. The purpose of stability testing is to provide evidence on how the quality of a drug substance or drug product varies with time under the influence of a variety of environmental factors such as temperature, humidity, and light; it enables recommendation of storage conditions, retest periods, and shelf lives to be established. Regulatory agencies recommend the use of stability-indicating methods for the analysis of stability samples. Thus, stress studies are required in order to generate the stressed samples, method development and method validation [3]. Forced degradation of CPS was performed under stress conditions (acid, alkaline, photolytic, thermal and oxidative), to establish the stability indicating nature of the method, and stressed samples were analyzed by the proposed method. The proposed RP-HPLC method was validated by assessing its specificity, linearity, accuracy, precision, limits of detection and quantification, system suitability parameters, ruggedness and robustness.

Many researchers reported methods for the assay of cefpirome in pharmaceutical formulations and in human serum [4–9]. These methods involve either complicated mobile phases or buffers that may be corrosive to the column or flow system of HPLC. Previous investigators [10–13] have also involved various microbiological assay methods, including both microdilution and agar dilution, to quantitate cefpirome. Two studies [13, 14] reported the pharmacokinetics of cefpirome in adults after quantitation of the compound in serum by using high-performance liquid chromatography (HPLC) assay for which specific details were not provided.

## Experimental

### Chemicals and Reagents

Gift sample of CPS was received from Alkem Labalories, Mumbai, India. HPLC grade methanol was purchased from Rankem, India. Hydrogen peroxide was purchased from Qualigens Fine chemicals, India and sodium hydroxide was purchased from Merck Ltd. India. High pure water was prepared by using Millipore Milli Q plus puri cation system. Commercial formulations Bacirom® (vial) and Forgen® (vial) containing 250mg of CPS were purchased from the local market.

### HPLC instrumentation and conditions

Quantitative HPLC was performed on Shimadzu HPLC with LC 10 AT VP series pumps besides SPD 10 A VP UV-Visible detector. The chromatographic separations were performed using LiChroCART-Lichrosphere100, C18, RP column (250 mm × 4mm × 5 μm) maintained at ambient temperature, eluted with mobile phase at a flow rate of 1ml/min for 10 min. The mobile phase consisted of methanol-water (50:50 % v/v). Measurements were made with injection volume 20μl and ultraviolet (UV) detection at 270 nm.

### Standard and Sample Preparation

The standard stock solution of CPS (1mg/ml) was prepared by dissolving 25 mg CPS in 25 ml volumetric flask containing 10 ml of methanol and 10 ml of water. The solutions was sonicated for about 10 min and later diluted to desired volume with mobile phase. Standard calibration solutions of CPS having concentration in the range of 0.5–200μg/ml were prepared by diluting stock solution with mobile phase.

From the vial equivalent to 25 mg of CPS content was transferred into a 25ml volumetric flask containing 15 ml mixture of methanol and water, ultrasonicated for 15 min, and then diluted up to the mark with mobile phase to yield sample stock solution. A small portion of sample solution was filtered through 0.45μm filter paper and used for injection on HPLC.

### Procedure for forced degradation study

Forced degradation of each drug substance and the drug product was carried out under acid, alkaline, photolytic, thermal and oxidative stress conditions. Acid degradation of drug substance and drug product in solution state was conducted with 0.1N hydrochloric acid for 48 h. Before injecting to HPLC system the samples were neutralized with 0.1N sodium hydroxide solution and made up to volume with mobile phase. Alkaline degradation of drug substance and drug product in solution state was conducted with 0.1N sodium hydroxide for 48 h. Before injecting to HPLC system the samples were neutralized with 0.1N hydrochloric acid solution and made up to volume with mobile phase. For photolytic stress, sample solutiosn of drug substance and drug product, were kept for 24 h in direct sunlight. For thermal degradation study, the sample solutions were kept in water bath for 2 h at 80°C, and after exposure to heat it was cooled to room temperature. For oxidative stress, sample solutions of drug substance and drug product of CPS, in 3% hydrogen peroxide were kept at ambient temperature for 48 h. Before injecting to HPLC system the samples were made up to volume with mobile phase. From these solutions, 20 μl were injected separately in HPLC system to obtain chromatograms.

## Results and Discussion

### Optimization of the Method

The proposed RP-HPLC method utilizes a LiChroCART-Lichrosphere100, C18 RP column (250 mm × 4mm × 5 μm) in an isocratic separation mode with mobile phase methanol and water in the proportion of 50:50 % (v/v), at a flow rate 1ml/min and the effluent was monitored at 270 nm. The retention time of CPS was 2.733 min. Degradation products resulting from the stress studies did not interfere with the detection of CPS, and the assay is thus stability-indicating.

### Results of forced degradation studies

**Fig. 2:**
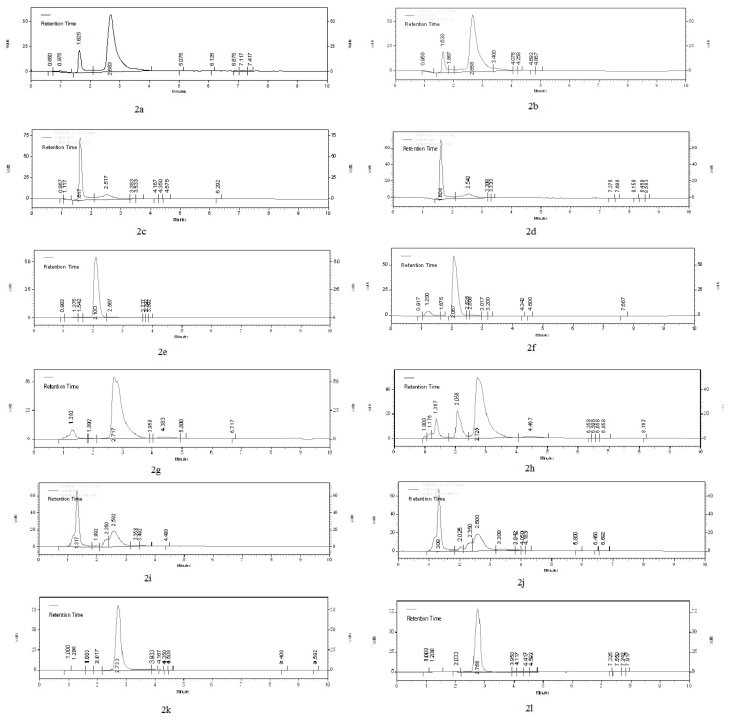
Representative chromatogram a) Pure CPS (10μg/ml) in acidic SC b) CPS (formulation) in acidic SC c) Pure CPS (10μg/ml) in alkaline SC d) CPS (formulation) in alkaline SC e) Pure CPS (10μg/ml) in oxidative SC f) CPS (formulation) in oxidative SC g) Pure CPS (10μg/ml) in photolytic SC h) CPS (formulation) in photolytic SC i) Pure CPS (10μg/ml) in thermal SC j) CPS (formulation) in thermal SC k) A Typical Chromatogram of CPS (10μg/ml) in pure form l) A Typical Chromatogram of CPS (10μg/ml) in Formulation SC…stress condition

Much degradation was observed in CPS samples under all stress conditions such as acid (fig. 2a and 2b), alkaline hydrolysis (fig. 2c and 2d), oxidative (fig. 2e and 2f), photolysis (fig. 2g and 2h) and thermal degradation (fig. 2i and 2j). Table 1 indicates the extent of degradation of CPS under various stress conditions. Drug degradation was observed when CPS was treated with mild alkali (in 0.1N NaOH for 48 h.) and a new degradant was eluted at 1.6 min. When exposed to direct sun light for 24 h. its degradants show different peaks at 1.2 min, 4.383 min, which indicate that CPS is sensitive to light. Under oxidation, one major degradant and some unknown degradation products were formed (Fig. 2e and 2f). The pure as well as formulation were showing total degradation under oxidative stress testing. When CPS was treating with 80°C of temperature for 2 h it was degrade and some new peaks were formed. Assay of CPS was unaffected by the presence of other degradants which confirms the stability-indicating power of the method.

### Method validation

The described method has been validated for linearity, precision, accuracy, specificity, LOD and LOQ, system suitability parameters, ruggedness and robustness.

### Linearity

Least square regression analysis was carried out for the slope, intercept and correlation coefficient (Table 2). The linear fit of the system was illustrated graphically. The linearity range was found to be 0.5–200μg/ml. Regression equation for CPS was

$$\text{y}=24341\text{x}+4144.8\;({\text{R}}^{2}=0.9999)$$

### Accuracy

This experiment was performed at three levels in which sample stock solutions were spiked with standard drug solution containing 80, 100 and 120% of labeled amount of the drugs (250 mg CPS) in vial. Three replicate samples of each concentration level were prepared and the % recovery at each level (n = 3), and mean % recovery (n=9) were determined (Table 3). The mean recovery was 99.46 %.

### Precision

The precision of the proposed method was evaluated by carrying out eight independent (50μg/ml) assays of test sample. RSD (%) of eight assay values obtained was calculated. Intermediate precision was carried out by analyzing the samples by a different analyst on another instrument. F-test and t-test were applied to the two sets of data at 95% confidence level, and no statistically significant difference was observed. The resultant data was presented in table 4.

### Specificity

Specificity is the ability to measure accurately and specifically the analyte of interest in the presence of other components that may be expected to be present in the sample matrix. It was found that the proposed method was specific because there is no interference of other active ingredients and excipients, ensuring that the peak response is due only to a single component. Based on the results, obtained from the analysis of forced degraded samples using the described method, it can be concluded that the method is specific for estimation of CPS in presence of degradants.

### LOD and LOQ

The detection and quantification limits were evaluated from calibration curves plotted in concentration range of 0.5–200μg/ml. The acceptance criterion for these replicate injections was RSD not more than 30% for LOD concentration and not more than 10% for LOQ concentration. The formulae used were LOD= 3.3σ/S and LOQ= 10σ/S (where σ = standard deviation of response and S = slope of calibration curve). The standard drug solutions for each value of LOD and LOQ concentration were injected 5 times. % RSD values for the area of replicate injections were calculated. LOD and LOQ for this method were found to be 0.110 and 0.364, respectively. These values indicated the method was very sensitive to quantify the drug.

### System suitability parameters

System suitability parameters can be defined as tests to ensure the method can generate results of acceptable accuracy and precision. The system suitability parameters like Theoretical plates (N), Resolution (R), and Tailing factor (T) were calculated and compared with the standard values to ascertain whether the proposed RP-HPLC method for the estimation of CPS in pharmaceutical formulations was validated or not. System suitability is usually developed after method development and validation has been completed. The obtained value of Theoretical plates (N) in this method was 4369, and the tailing factor was found to be 1.224.

### Robustness

The percentage recovery of CPS was good under most conditions and did not show any significant change when the critical parameters were modified. The tailing factor was always less than 2.0, and the components were well separated under all the changes carried out. Thus the method conditions were robust.

### Assay

The validated method was applied to the determination of CPS in commercially available Bacirom® (vial) and Forgen® (vial). Figure 2k-2l illustrates two typical HPLC chromatograms obtained from CPS standard solution and from the assay of Bacirom®. The observed concentration of CPS was found to be 247.67±0.271mg (mean±SD) for Bacirom® and 247.95±0.08 for Forgen®. The results of the assay (*n* = 9) undertaken yielded 99.06 % (%RSD = 0.1) of label claim for CPS in Bacirom® and 99.18 % (%RSD = 0.032) of label claim for CPS in Forgen®. The retention times of CPS were found to be 2.733 and 2.758 for standard drug and formulation, respectively. The results of the assay indicate that the method is selective for the estimation of CPS without interference from the excipients used to formulate and produce these tablets.

## Conclusion

The LC method described here is a very simple, sensitive, and accurate procedure for estimation of CPS. The developed and validated LC method is stability-indicating and enables specific, accurate, robust and precise analysis of CPS in formulations. The method is sensitive enough for quantitative detection of the analyte in pharmaceutical preparations. The proposed method can thus be used for routine analysis, quality control and for studies of the stability of pharmaceutical tablets containing these drugs. The validation data indicate good precision, accuracy and reliability of the method. The sample recoveries in all formulations were in agreement with their respective label claims, and they suggested non-interference of formulation excipients in the estimation.

## Figures and Tables

**Tab. 1:** Results of analysis of forced degradation study

Stress condition/duration	CPS	
	
	% Recovery	Retention Time
**Acid degradation**		
RS (0.1N HCL/48 h)	84.352	2.683
Formulation (0.1N HCl/48 h)	81.903	2.658

**Alkaline degradation**		
RS (0.1N NaOH/48 h)	70.717	1.617
Formulation (0.1N NaOH/48 h)	73.562	1.608

**Photolysis**		
RS (Sun light/24 h)	87.403	2.717
Formulation (Sun light/24 h)	70.925	2.725

**Thermal degradation**		
RS (water bath/2 h at 80°C)	53.350	1.317
Formulation (water bath/2 h at 80°C)	52.229	1.308

**Oxidative degradation**		
RS (3%w/v/48 h)	96.982	2.100
Formulation (3%w/v/48 h)	87.791	2.067

RS…reference standard.

**Tab. 2:** Regression characteristics of the proposed HPLC method

Linearity experiment (n=5)	CPS
Range (μg/ml)	0.5–200
Mean ‘R’ value	0.9995
Slope	24341
Intercept	4144.8

**Tab. 3:** Results of Accuracy experiment using proposed method

	CPS		
	
	Taken(μg)	Recovered (μg)	% Recovery
LEVEL 1(80)	8	7.96	99.53
LEVEL 2(100)	10	9.96	99.64
LEVEL 3(120)	12	11.90	99.23

Mean % recovery (n=9)		99.466±0.254	

%RSD		0.253	

**Tab. 4:** Results of Precision study

Precision	CPS
	
	Mean assay (%)/%R.S.D
Set 1(n=5)	99.8/0.929
Set 2(n=5)	99.6/0.871

	**Calculated value/critical value**

F-test	0.920/3.368
t-test	0.427/2.106

## References

[1] Block JH, Beale JM (2004). Wilson and Gisvold’s Textbook of Organic Medicinal and Pharmaceutical Chemistry.

[2] Turley CP, Kearns GN, Jacobs RF (1988). Microanalytical high-performance liquid chromatography assay for cefpirome (HR 810) in serum. Antimicrob Agents Chemother.

[3] International Conference on Harmonization (ICH) (2003). Topic Q1A (R2). Stability testing of new drug substances and products.

[4] Breilh D, Lavallee C, Fratta A, Ducint D, Makhoul PC, Saux MC (1999). Determination of cefepime and cefpirome in human serum by high-performance liquid chromatography using an ultrafiltration for antibiotics serum extraction. J Chromatogr B Biomed Sci Appl.

[5] Uihlein M, Klesel N, Seeger K (1988). Determination of cefpirome (HR 810) in serum and urine. Infection.

[6] Sugioka T, Asano T, Chikaraishi Y, Suzuki E, Sano A, Kuriki T (1990). Stability and degradation pattern of cefpirome (HR810) in aqueous solution. Chem Pharm Bull.

[7] Ip M, Au C, Cheung SW, Chan CY, Cheng AFB (1998). A rapid high-performance liquid chromatographic assay for cefepime, cefpirome and meropenem. J Antimicrob Chemother.

[8] Sriwiriyajan S, Mahatthanatrakul W (2010). Development of an analytical method for cefpirome in plasma by simplified HPLC technique and its applications. Arzneimittelforschung.

[9] Arayne MS, Sultana N, Nawaz M (2008). A RP-HPLC method for the assay of Cefpirome and its application in drug-metal interaction studies. J Anal Chem.

[10] Bertran MA, Bruckner DA, Young LS (1984). In vitro activity of HR 810, a new cephalosporin. Antimicrob Agents Cemother.

[11] Jones RN, Thornsberry C, Barry AL, Ayers L, Brown S, Daniel J, Fuchs PC, Gavan TL (1984). Disk diffusion testing, quality control guidelines, and antimicrobial spectrum of HR 810, a fourth-generation cephalosporin, in clinical microbiology laboratories. J Clin Microbiol.

[12] Seibert G, Limbert M, Winkler I, Dick T (1983). The antibacterial activity in vitro and β-lactamase stability of the new cephalosporin HR 810 in comparison with five other cephalosporins and two aminogycosides. Infection.

[13] Maass L, Malerczyk V, Verho M, Hajdu P, Seeger K, Klesel N (1987). Dose linearity testing of intravenous cefpirome (HR 810), a novel cephalosporin derivative. Infection.

[14] Malerczyk V, Maass L, Verho M, Hajdu P, Klesel N, Rangoonwala R (1987). Single and multiple dose pharmacokinetics of intravenous cefpirome (HR 810), a novel cephalosporin derivative. Infection.

